# *Leishmania* infection in cats and dogs housed together in an animal shelter reveals a higher parasite load in infected dogs despite a greater seroprevalence among cats

**DOI:** 10.1186/s13071-020-3989-3

**Published:** 2020-03-20

**Authors:** Gad Baneth, Yaarit Nachum-Biala, Adam Zuberi, Nirit Zipori-Barki, Laor Orshan, Gabriela Kleinerman, Ayelet Shmueli-Goldin, Michel Bellaiche, Monica Leszkowicz-Mazuz, Harold Salant, Daniel Yasur-Landau

**Affiliations:** 1 Koret School of Veterinary Medicine, The Hebrew University of Jerusalem, Rehovot, Israel; 2 The Israeli Veterinary Services and Animal Health, Israeli Ministry of Agriculture, Bet Dagan, Israel; 3 Central Laboratories Jerusalem, The Israeli Ministry of Health, Jerusalem, Israel; 4 Division of Parasitology, Kimron Veterinary Institute, Bet Dagan, Israel

**Keywords:** High resolution melt analysis PCR, ITS1 PCR, Cat, Dog, Parasite load, *Leishmania infantum*

## Abstract

**Background:**

An outbreak of leishmaniosis was studied in cats and dogs housed together with no separation in an animal shelter in Israel.

**Methods:**

The study included recording of clinical signs, serology for *Leishmania* infection by ELISA, PCR of blood for *Leishmania* DNA by ITS1 HRM and kDNA PCR, parasite quantification, and trapping of sand flies around the shelter.

**Results:**

Thirty-seven % (22/60) of the dogs and 75% (50/67) of the cats were seropositive to *L. infantum* with a significantly higher seropositivity rate in the cat population (*χ*^2^ = 42.160, *P* < 0.0001). Twenty-five percent (15/60) of the dogs were positive for *Leishmania* by blood PCR, 12% by the *Leishmania* ITS1 HRM PCR and 22% by kDNA PCR. Of the cats, 16% (11/67) were positive by kDNA PCR and none by ITS1 HRM PCR. All the PCR-positive animals were infected by *L. infantum* verified by DNA sequencing and there was no significant difference between the PCR-positivity in the dog and cat populations. Altogether, 43% (26/60) of the dogs and 79% (53/67) of the cats were positive by serology or PCR for *L. infantum*. The average *Leishmania* parasite load in the blood of PCR-positive dogs (42,967 parasites/ml) was significantly higher than in PCR-positive cats (1259 parasites/ml) (*t*_(12)_ = 2.33, P = 0.037). Dogs that were positive by the *Leishmania* ITS1 HRM PCR and kDNA PCR had significantly higher parasite loads than dogs positive only by the kDNA PCR (*t*_(11)_ = − 3.186580, *P* < 0.009). No significant effect was found for FIV seropositivity on *Leishmania* infection in the cats (*χ*^2^ = 0.506, *P* = 0.777). A higher percentage of *Leishmania*-positive dogs showed clinical signs compatible with leishmaniosis compared to *Leishmania*-positive cats (100 *vs* 52.8%, *χ*^2^ =15.242, *P* < 0.0001). *Phlebotomus perfiliewi*, a proven vector of *L. infantum*, comprised 92% of trapped sand flies.

**Conclusions:**

Comparisons of populations of cats and dogs exposed to sand flies and *L. infantum* under the same conditions indicated that although a high rate of exposure was detected in cats as manifested by a significantly greater degree of seropositivity, dogs had significantly higher blood parasite loads, and were likely to be more infectious to sand flies than cats.
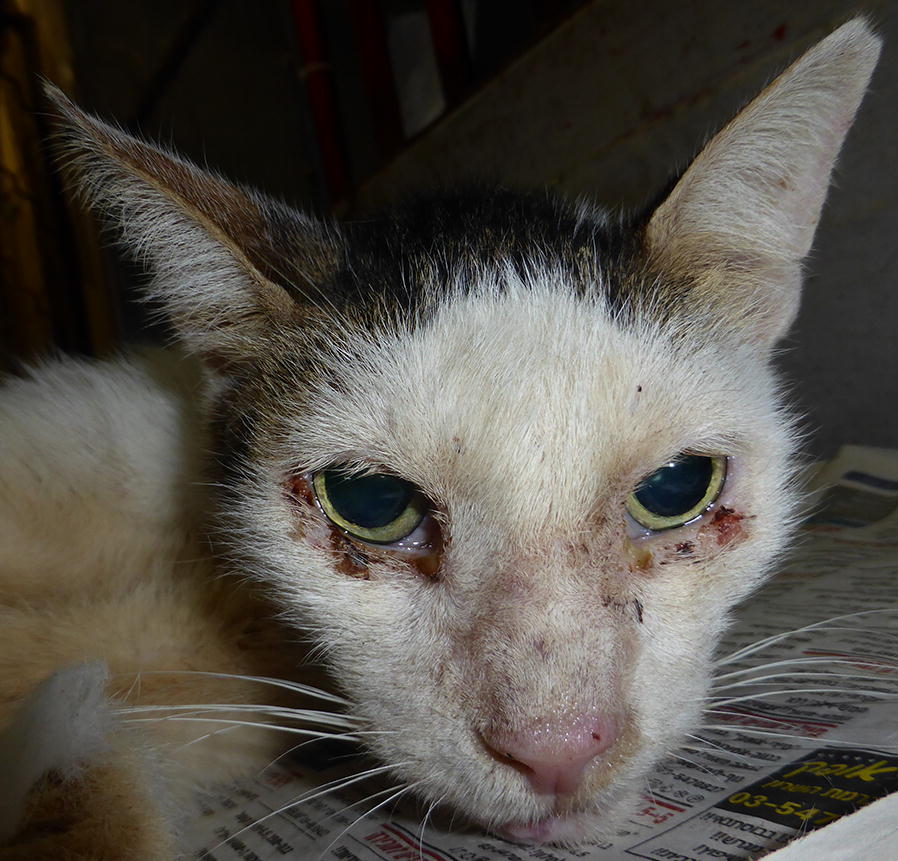

## Background

Leishmaniosis caused by *Leishmania infantum* is a major zoonosis which affects humans and dogs in many regions of the world [1]. Cats also develop clinical disease due to *L. infantum* infection and may have dermal as well as visceral manifestations of this infection [2, 3]. Although the number of feline cases of clinical disease with *L. infantum* seems lower than the number of canine cases in endemic areas, there is no sufficient comparative knowledge relating to the prevalence of leishmaniosis in domestic dogs and cats exposed to the same conditions of disease transmission.

Infection and disease with *L. infantum* may be related to several factors including biting preferences of sand flies, the size of inoculum during the sand fly bite, number of exposures to infectious bites during the sand fly season, the hostʼs individual susceptibility, the nature of the immune response mounted to infection, co-infection with other pathogens and possible host immune suppression.

This study investigated an outbreak of leishmaniosis in cats and dogs housed together with no separation in an animal shelter in Israel. It provided a unique opportunity to evaluate infection in dog and cat populations exposed to the same environmental and physical conditions.

## Methods

### Dogs and cats included in the study

Totals of 67 cats and 60 dogs housed together in a private animal shelter in northern Israel were included in the study. The animals had free movement within the compound and there was no separation between cats and dogs. The shelter was eventually closed by the Israeli Veterinary Services due to poor sanitary and nutritional conditions and the animals were moved in June 2018 to temporary accommodation where they all had a physical examination and collection of blood samples to test for leishmaniosis due to the presence of suspicious skin lesions in some of the dogs and cats.

### Collection of blood

Blood was collected by venipuncture of the jugular or cephalic veins of the dogs and cats who were included in the study on June 25th 2018. The blood was collected into EDTA and clot tubes for PCR and *Leishmania* serology, respectively. Physical examination was performed by experienced veterinarians and abnormalities were recorded for each animal.

### Serology

Serum anti-leishmanial antibodies were determined by ELISA, using crude leishmanial antigen, essentially as previously described [4]. All sera were diluted to 1:100 and incubated with leishmanial antigen (*L. infantum* strain MCAN/IL/2010/TR1) coated plates for one hr at 37 °C. The plates were then washed with 0.1% Tween 20 in 50 mM phosphate-buffered saline (PBS) at pH 7.2. Cat sera were then incubated with rabbit anti cat IgG antigen conjugated to horseradish peroxidase (1:10,000 dilution; OriGene Technologies, Inc. Rockville, MD, USA) whereas dog sera were incubated with Protein A conjugated to horseradish peroxidase (1:10,000 dilution; Zymed Laboratories, Inc., San Francisco, CA, USA) for 1 hr at 37 °C. Excess conjugate was removed by extensive washing in PBS-Tween and the plates were developed by addition of the substrate 2,29-azino-di-3-ethylbenzthiazoline sulfonate (ABTS) (Sigma-Aldrich-Merck, Jerusalem, Israel). Each plate was read when the absorbance (lambda = 405 nm) of the positive reference serum reached an optical density (OD) value between 1.1–1.2 for cat samples and 1.2–1.4 for dog samples. A titration of positive and negative reference cat or dog sera were included on each plate to monitor inter-assay variation. A serological cut-off of 0.4 OD for cats and for dogs was calculated based on four standard deviations above the mean OD values of readings from eight control sera of PCR-negative cats and dogs, respectively, from non-endemic areas for leishmaniosis. Borderline positive levels were considered antibody levels that were in the range between the three and four standard deviations which were 0.2–0.4 OD for dogs and 0.3–0.4 for cats.

Serology of the cat sera for antibodies against the feline immunodeficiency virus (FIV) was performed using the Megacor *FAST*est (Megacor Diagnostik, Hoerbranz, Austria).

### DNA extraction, PCR for *Leishmania* and DNA sequencing

DNA was extracted from 200 µl of EDTA-anticoagulated blood samples from the dogs using the Illustra blood genomicPrep Mini Spin Kit (GE Health care, Buckinghamshire, UK), following the manufacturer’s instructions. *Leishmania* detection was performed by real-time PCR using primers JW11/JW12 targeting a 120 bp sequence of the *Leishmania* short fragment from the kinetoplast minicircle (kDNA) [5]. Additional detection and identification was carried out by PCR using primers ITS-219F and ITS-219R to amplify a 265-bp fragment of the *Leishmania* ribosomal operon internal transcribed spacer 1 (ITS1) region and then evaluated by high resolution melt (HRM) analysis (*Leishmnia* ITS1 HRM PCR) [6]. PCR was performed using the StepOnePlus real-time PCR thermal cycler (Applied Biosystems, Foster City, CA, USA) as previously described [7]. DNA extracted from parasite promastigote culture of *L. infantum* was used as positive control for PCR and DNA from colony-bred dogs negative by PCR for vector-borne pathogens including *L. infantum* was used as a negative control. A non-template control (NTC) with the same reagents described above but without DNA was added to each PCR to rule out contamination.

Positive DNA amplicons were purified (EXO-Sap, New England Biolabs Inc., Ipswich, MA, USA) and sequenced in the Center for Genomic Analyses at the Hebrew University (Jerusalem, Israel) using the BigDye Terminator cycle from Applied Biosystems ABI3700 DNA Analyzer. The ABI Data Collection and Sequence Analysis software (ABI, Carlsbad, CA, USA) was used for analysis. DNA sequences were compared to other sequences deposited in GenBank using the BLASTn website hosted by NCBI, National Institutes of Health, USA (http://www.ncbi.nlm.nih.gov).

### Quantitation of *Leishmania* in blood

Samples that were positive by PCR were further analyzed to evaluate the blood parasite load. Quantitative PCR of leishmanial DNA from the positive samples was carried out by amplification of a 120 bp of the kDNA using primers JW11/JW12 [5]. To generate a standard curve, 10-fold dilutions of DNA extracted from parasite promastigote cultures of *L. infantum* at concentrations of 10^1^–10^8^ promastigotes were used. Real-time PCR was carried as mentioned above. The number of parasites in each sample was calculated against the standard curve using the StepOne software version 2.2.2 (Thermo Fisher Scientific, Waltham, MA USA).

### Sand flies

Sand flies were collected from five locations around the fence of the animal shelter on July 30th 2018. Modified CDC traps operated without light powered by two 1.2V AA rechargeable batteries and baited with one kg of dry ice were placed from sunset to sunrise in a vertical position. The openings were parallel to the ground and ~ 10 cm above it and the fan was adjusted to create updraft airflow with the collection boxes hanging above the body of the trap. The catch was chilled and kept at − 20 °C until sorting. All sand fly males were identified to the species level by the morphology of the genitalia using the keys of Abonnec & Lewis [8, 9]. The females were not identified to the species level and pooled in groups of 20 specimens for molecular detection of *Leishmania* DNA. All samples were stored at − 20 °C until testing. DNA was extracted from the homogenized pooled females using the QIAsymphony DNA Mini Kit and the QIAsymphony SP machine (Qiagen N.V. Venio, The Netherlands) and PCR was performed using the *Leishmania* ITS1 HRM PCR [6]. PCR was performed in a total volume of 20 μl, containing 10 μl of AccuMeltHRM SuperMix (Quanta Bioscience, Gaithersburg, USA), 0.5 μM of each primer, 5% DMSO (w/v) and 3 μl DNA, using the Roche LightCycler 96^®^ (Roche, Mannheim, Germany). The cycling parameters were 95 °C for 5 min; 45 cycles of 95 °C for 10 s; 60 °C for 45 s, 95 °C for 60 s, 40 °C for 60 s, 65 °C for 1 s 97 °C for 1 s, 37 °C for 30 s.

### Statistical analysis

Student’s t-test and the Chi-square test were used for ordinal (positive, negative and borderline) and nominal (parasites load) variables, respectively. The continuity correction was used when 2 × 2 tables were used. Pearsonʼs correlation coefficient was used to test correlation between variables. Statistical analysis was performed using the statistics software SPSS(R) 25.0 software (IBM, Armonk, New York, USA). Statistical significance was defined as *P* < 0.05.

## Results

### Serology, PCR and parasite loads

Thirty-seven percent (22/60) of the dogs were seropositive to *L. infantum* antigen by ELISA whereas 75% (50/67) of the cats were seropositive (Table 1). Accordingly, seroreactivity with *L. infantum* in the cat population at the shelter was significantly higher than in the dog population (*χ*^2^ = 42.160, *df* = 1, *n* = 127, *P* < 0.0001).

**Table 1:** Results of ELISA serology and PCR in dogs and cats from the animal shelter

Diagnostic assay	Animal status	Dogs *n* (%)	Cats *n* (%)
Serology	Positive	22 (36.6)	50 (74.6)
	Borderline	4 (6.6)	14 (20.9)
	Negative	34 (56.6)	3 (4.5)
	Total	60	67
PCR (*Leishmania* ITS1 HRM and kDNA)	Positive	15 (25.0)	11 (16.4)
	Negative	45 (75.0)	56 (83.6)
	Total	60	67
Positive by serology and PCR		11 (18.3)	8 (11.9)
Positive by serology and/or PCR		26 (43.3)	53 (79.1)

Twenty-five percent (15/60) of the dogs were positive for *Leishmania* by blood PCR. Seven (12%) were positive by the *Leishmania* ITS1 HRM PCR and 13 (22%) by kDNA PCR, with five positive by both PCRs, eight only by kDNA and two only by ITS1 PCR. Of the cats, 16% (11/67) were positive by kDNA PCR and none were positive by ITS1 PCR. All the PCR-positive animals were verified as positive to *L. infantum* by DNA sequencing and BLAST analysis (Additional file 1: Table S1). There was no significant difference between the PCR-positivity prevalence of the dog and cat populations (*χ*^2^ = 1.445, *df* = 1, *n* = 127, *P* = 0.229).

Altogether, 43% (26/60) of the dogs and 79% (53/67) of the cats were positive either by serology or PCR for *L. infantum* and the prevalence of positivity by at least one of these diagnostic methods in the cats was significantly higher than in the dog population (*χ*^2^ = 39.221, *df* = 1, *n* = 127, *P* < 0.0001). Of the PCR-positive dogs, 11/15 were seropositive (OD > 0.4), two were borderline positive (OD 0.2–0.4) and two were seronegative (OD < 0.2) (Table 1). All the PCR-positive cats were either seropositive (OD > 0.4; *n* = 8) or borderline-positive (OD 0.3–0.4; *n* = 3).

The average *Leishmania* parasite load in the blood of PCR-positive dogs (42,967 parasites/ml; range: 305–219,975) was significantly higher than the parasite load in PCR-positive cats (1259 parasites/ml; range: 470–1975) (*t*_(12)_ = 2.33, *P* = 0.037) indicating that PCR-positive infected dogs harbored a higher parasite load in their blood than PCR-positive infected cats exposed to *L. infantum* infection under the same environmental conditions. Furthermore, while there was a fair correlation (*r*^2^ = 0.7, *P* = 0.007) between the antibody levels expressed as OD in dogs and their blood parasite load, there was no such positive correlation found between antibody levels and parasite loads for the PCR-positive cats (*r*^2^ = − 0.125, *P* = 0.73). Dogs that were positive by the *Leishmania* ITS1 HRM PCR and kDNA PCR (*n* = 5) had significantly higher parasite loads than dogs that were positive only by the kDNA PCR (*n* = 8) (averages 97,157 parasite/ml and 9099 parasite/ml, respectively (t_(11)_ = − 3.186580, *P* = 0.009).

FIV serology was positive in 7% (5/67) of the cats. Of these cats, two were seropositive for *L. infantum*, one was borderline seropositive, and two were seropositive and PCR-positive. No significant effect was found for FIV seropositivity on *Leishmania* infection in the evaluated cats (*χ*^2^ = 0.506, *df* = 1, *n* = 67, *P* = 0.777).

**Fig. 1:**
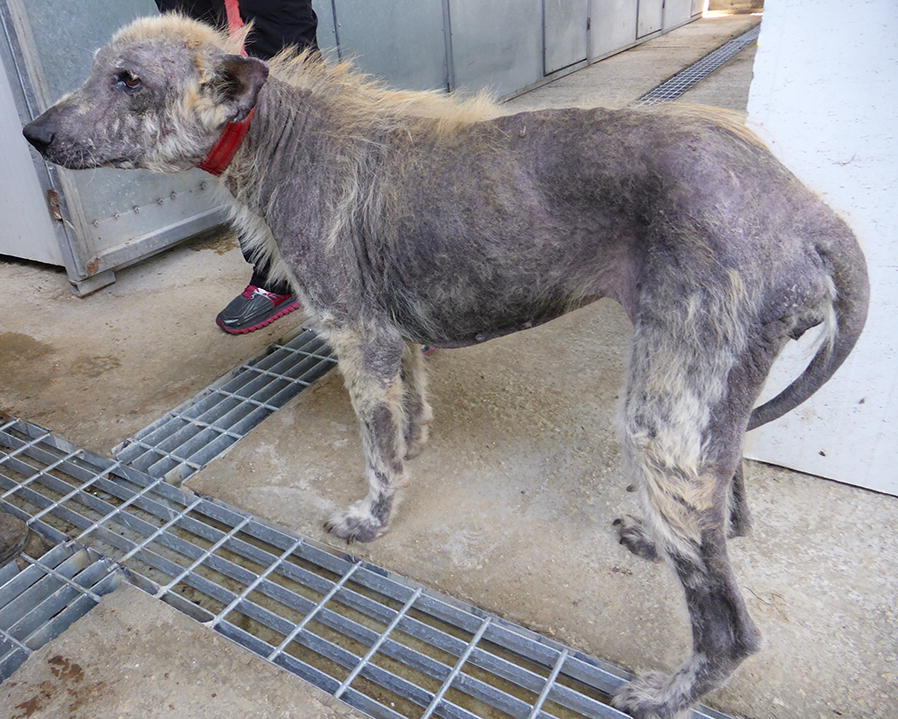
A dog from the animal shelter that was positive for *L. infantum* by serology and PCR. The dog has overt alopecia and hyperkeratosis with purulent keratoconjunctivitis typical of canine leishmaniosis

**Fig. 2:**
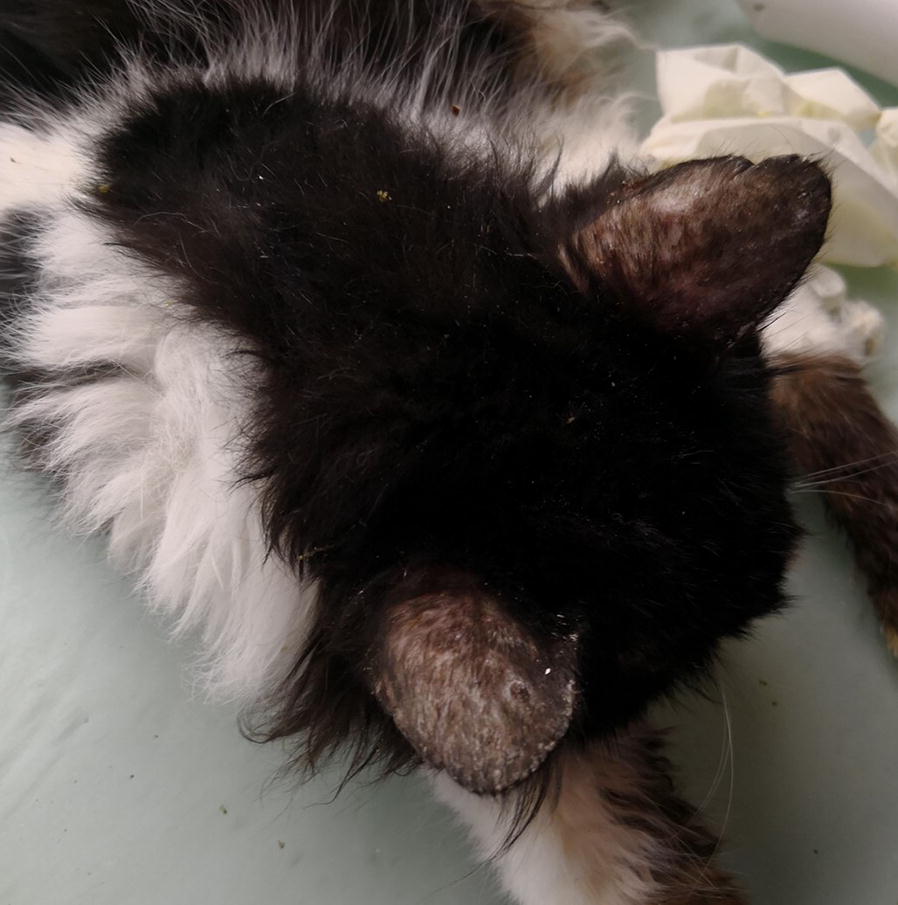
Aural alopecia and in a cat from the animal shelter that was seropositive with a high antibody level for *L. infantum*

**Fig. 3:**
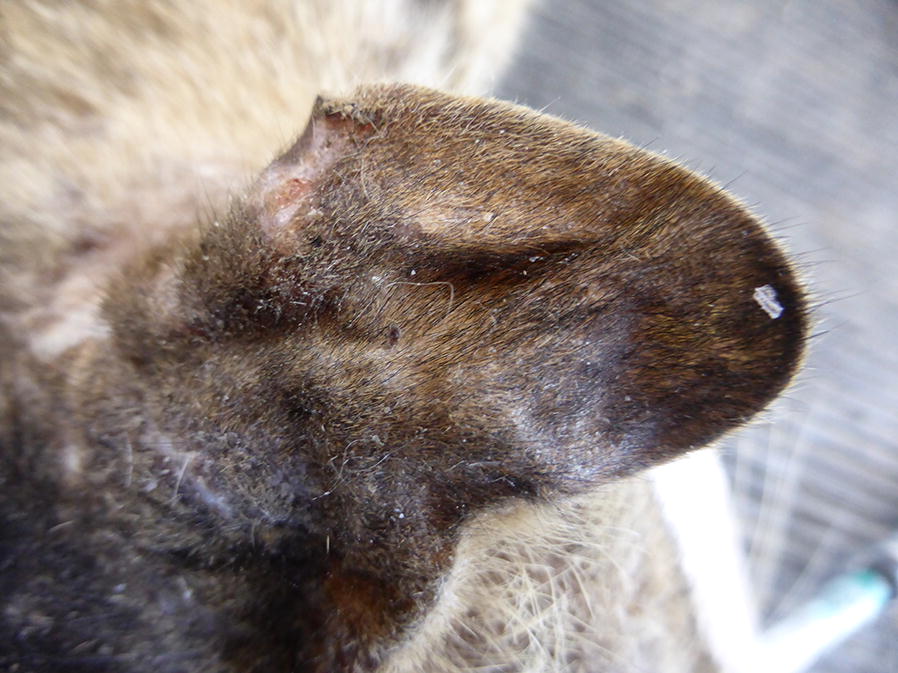
Ulcerative skin lesions, partial alopecia and scales on the ear of a cat from the animal shelter that was seropositive with a high antibody level for *L. infantum*

**Fig. 4:**
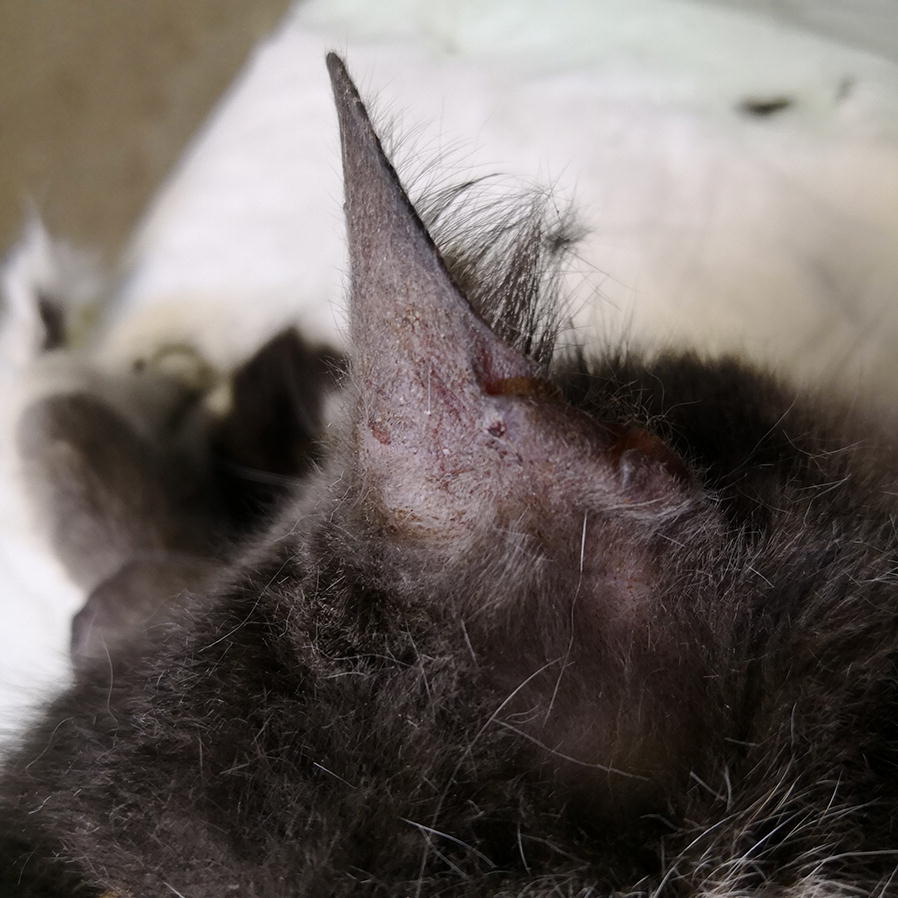
Complete alopecia and skin ulceration in the ear of a *L. infantum-*seropositive cat with a high antibody level

All dogs and cats were screened for clinical signs on physical examination. Clinical signs potentially related to leishmaniosis included skin lesions (Figs. 1, 2, 3, 4), lymphadenomegaly, splenomegaly, ocular lesions, severe weight loss and emaciation. These clinical signs may have also been associated with some other medical conditions which were not determined due to the limited resources devoted for the medical examination of the animals. Overall, 88% (53/60) of the dogs had clinical signs that could be associated with leishmaniosis, nevertheless, only 49% (26/53) were positive for *Leishmania* infection by serology or PCR. Fifty-five percent (37/67) of the cats had clinical signs compatible with leishmaniosis, of which 76% (28/37) were positive by serology or PCR for *L. infantum*. All of the dogs that were positive by either serology or PCR had clinical signs compatible with leishmaniosis, but only 53% (28/53) of the cats which were positive by at least one of these diagnostic techniques had clinical signs of disease. Overall, a higher percentage of *Leishmania*-positive dogs showed clinical signs that could be related to leishmaniosis compared to *Leishmania*-positive cats (100 *vs* 53%, *χ*^2^ = 15.242, *df* = 1, *n* = 127, *P* < 0.0001).

### Sand flies

A total of 633 *Phlebotomus* spp. sand flies were collected by the five traps at the shelter approximately 8 weeks after the animals had been removed from it (Table 2). *Phlebotomus perfiliewi*, a proven vector of *L. infantum*, comprised 92% of trapped sand flies, while *P. tobbi* and *P. papatasi* accounted for 3% and 5% of the remaining sand flies, respectively*. Leishmania* DNA was not detected in any of the 19 female sand fly pools tested.

**Table 2:** Trapping of sand flies in the shelter area and identification of sand fly species

Trap No.	No. of specimens	No. of females (%)	No. of males (%)	No. of sand fly pools tested for *Leishmania* DNA	No. of male *Phlebotomus perfiliewi* (%)^a^	No. of male *Phlebotomus papatasi* (%)^a^	No of male *Phlebotomus tobbi* (%)^a^
1	289	235 (81)	54 (19)	8	52 (96)	0	2 (4)
2	90	45 (50)	45 (50)	3	45 (100)	0	0
3	68	32 (47)	36 (53)	2	36 (100)	0	0
4	118	64 (54)	54 (46)	3	46 (85)	0	8 (15)
5	68	53 (78)	15 (22)	3	8 (53)	7 (43)	0
Total	633	429 (68)	204 (32)	19	187 (92)	7 (3)	10 (5)

^a^Percent of males

## Discussion

Most of the epidemiological surveys on canine and feline leishmaniosis have studied only dogs or cats, and very few have studied infection in both animal species living in the same area [10, 11]. The present study included dogs and cats that shared a small restricted compound where they mixed freely in the presence of abundant numbers of sand fly vectors, as inferred by the entomological survey performed after the shelter was evacuated from animals. It is reasonable to hypothesize that the dogs and cats living together in the same compound had similar exposure to infectious sand flies. Therefore, this study provided a unique opportunity to compare infection of dogs and cats with *L. infantum* which was different from previous studies performed in larger and more diverse environments such as metropolitan Lisbon and the Aeolian islands in Italy [10, 11].

Although serology and blood PCR tend to underestimate *L. infantum* infection rates in canine populations [12], and possibly also in feline populations, the present study detected high infection rates as indicated by serology and/or PCR in 43% of the dogs and 79% of the cats. These rates are higher than 41.8% and 25.8% found by serology, blood and conjunctival PCR for dogs and cats, respectively, in the small Aeolian islands of Lipari and Vulcano [11]. When evaluating only kDNA PCR of blood, 22% of the dogs and 16% of the cats in our study were positive while only 12.2% of the dogs and 2.1% of the cats in the Aeolian islands study were positive [11]. In contrast, in a study from the Lisbon metropolitan area in Portugal, 34.9% of the dogs and 20.3% of the cats were kDNA PCR-positive [10].

Surprisingly, seroreactivity with *L. infantum* in the feline population of the shelter (75%) was significantly more prevalent that in the canine population (37%). This is opposite from the situation in the Aeolian islands study where 25.7% of the cats and 34.6% of the dogs surveyed were positive by the indirect immunofluorescence test (IFAT) [11]. The ratio between cat and dog seroreactivity was even lower in the Lisbon metropolitan area study where only one cat of 76 was seropositive by IFAT whereas 20.4% of the dogs were seropositive, and the authors of the study suggested that the serological test which they used may not have been sufficiently sensitive for detection of anti-leishmanial antibodies in cats [10]. The reason for the high seroprevalence rate in the shelter cats from Israel may have been due to the extensive close contact of these cats with infected dogs harboring a high parasite load in the presence of abundant sand fly vectors in a small compound. It may have also been related to their close association with many other infected cats under these conditions.

The finding that *L. infantum* PCR-positive dogs had significantly higher parasite loads compared to PCR-positive cats exposed to infection under the same conditions agrees with the possibility that dogs may be better reservoirs and spreaders of *L. infantum* compared to cats. The parasite load in the blood and skin of dogs naturally infected with *L. infantum* has been found to be correlated with their capacity to infect sand fly vectors [13]. Although cats have been shown experimentally to infect sand flies by xenodiagnosis [14, 15], the relationship between their parasite loads and infectiousness to sand flies has not been studied, and it is unknown how successful they are as spreaders of *L. infantum* under natural conditions.

The fact that the ITS1 HRM PCR detected infection in dogs with significantly higher parasite loads than the dogs detected only by the kDNA PCR can be explained by the lower copy number of the ITS1 locus in comparison to the targeted copy number of kDNA minicircle sequence. There are about 10,000 copies of the kDNA and only 40–200 copies of the ITS1 region of the rRNA gene in each individual *Leishmania* parasite [16]. This also explains why cats in this study were negative by ITS1 HRM PCR despite being positive by kDNA, as their average blood parasite load was considerably lower than that of positive dogs. Hence, kDNA was shown to be preferred over the ITS1 as a *Leishmania* PCR target for blood PCR of cats.

The finding of a fair correlation between the antibody levels and parasite load in dogs but not in cats may also be associated with the relatively low parasite load of infected cats and perhaps the different biological behavior of *L. infantum* in exposed cats, where positive serology was highly prevalent, but the magnitude of blood parasitemia appeared to be lower than in dogs exposed to the same conditions.

FIV serology was positive in 7% of the cats and no statistically significant association was found between FIV and *L. infantum* infections. Some other studies on large numbers of cats in Italy and Brazil have found an association between feline leishmaniosis and FIV [17, 18] whereas studies from Spain and Cyprus failed to find such an association [19, 20]. It is likely that an association between FIV and *L. infantum* infection was not found in the present study due to the small number of FIV-positive cats detected in the shelter and the overall limited feline population size evaluated.

Evaluation of infection with other potential co-infecting agents such as *Ehrlichia canis*, *Anaplasma platys*, and *Babesia* spp. in dogs, and the feline leukemia virus (FeLV), *Bartonella* spp., hemotrophic mycoplasmas and *Toxoplasma gondii* infections in cats was not included in the study due to lack of financial support for the performance of these assays on samples from all the animals. Although FIV has been shown to be a common infection of shelter cats in Israel with 12% seroprevalence in a previous study, FeLV infection was found only in 4% of the cats in that study and seems to be an uncommon infection in Israeli cats [21].

The clinical evaluation of the cats and dogs was challenging because the animals were kept in poor sanitary and nutritional conditions at the shelter, and also due to the lack of further testing by hematology, serum biochemistry, and urinalysis which could provide important information on the clinical condition of infected animals [1, 2]. Furthermore, except for FIV serology, no other tests for additional infectious agents were performed. Most of the dogs (88%) and cats (55%) in the shelter had physical examination findings that were compatible with leishmaniosis and eventually after testing for the infection using specific tests, it was shown that indeed a significantly higher percentage of the dogs that were *Leishmania*-positive by serology or PCR had clinical signs of the disease when compared to their feline counterparts. This is reasonable when relating to the dog as the main host of the disease which often develops clinical disease after exposure to infection while the cat appears to be less frequently affected clinically by *L. infantum* with a much smaller number of clinical cases documented in the veterinary literature [2, 3].

To our knowledge, this is the first report of feline leishmaniosis in Israel. Israel is endemic for three species of *Leishmania*, *L. tropica* and *L. major*, which cause cutaneous leishmaniasis in humans, and *L. infantum* which causes visceral leishmaniasis [22]. Although *L. major* and *L. tropica* have been shown to cause disease in rare cases of dogs in Israel [23–25], and both of these species have been reported to infect cats apparently asymptomatically in Turkey [26, 27], infection in the animal shelter was shown to be caused by *L. infantum* by DNA sequencing of the PCR products.

*Phlebotomus perfiliewi* was the dominant species in the sand fly catch. This species is considered a proven vector of *L. infantum* in Algeria and Italy and putative vector in other regions around the Mediterranean Sea including Israel [28]. Its presence in relative high abundance in all the traps placed around the shelter indicates a high likelihood of local transmission of *L. infantum.* While the second most abundant sand fly species found in the shelter area, *P. papatasi* is a vector of *L. major* in Israel, but not of *L. infantum* [22], the third species *P. tobbi* found only in small numbers is a vector of *L. infantum* [29]. The lack of positive detection of *L. infantum* in the females is reasonable in a collection conducted eight weeks after the removal of the infected animals from the area, and low infection rates of *L. infantum* in sand flies in general [30, 31].

## Conclusions

Comparison of cats and dogs exposed similarly to sand fly vectors and *L. infantum* infection in a small compound revealed a higher degree of seropositivity in the cats with similar PCR-positivity rates in both species, however, a significantly higher parasite load in the dogs, suggesting that dogs could be more infectious to sand flies and efficient in spreading the infection.

## Supplementary information

**Additional file 1: Table S1.** Results of BLAST for *Leishmania* ITS1 and kDNA sequences amplified from the blood of dogs and cats included in the study by PCR. The GenBank accession number of first match by BLAST, species name, identity %, coverage %, and number of base pairs compared excluding the primer sequences are included for each positive animal.

## Data Availability

All data generated or analyzed during this study are included in this published article.

## Abbreviations

PCR: polymerase chain reaction
EDTA: ethylenediaminetetraacetic acid
NTC: non-template control
