# Supplementary material for: Leishmania infection in cats and dogs housed together in an animal shelter reveals a higher parasite load in infected dogs despite a greater seroprevalence among cats
Source: Parasit Vectors. 2020 Mar 20;13:115. doi: 10.1186/s13071-020-3989-3 (PMC7083040; doi:10.1186/s13071-020-3989-3)
Supplement: Supplementary file 1 — Additional file 1: Table S1. Results of BLAST for Leishmania ITS1 and kDNA sequences amplified from the blood of dogs and cats included in the study by PCR. The GenBank accession number of first match by BLAST, species name, identity %, coverage %, and number of base pairs compared excluding the primer sequences are included for each positive animal. [file 13071_2020_3989_MOESM1_ESM.docx]

**Additional file 1: Table S1**.

Results of BLAST for *Leishmania* ITS1 and kDNA sequences amplified from the blood of dogs and cats included in the study by PCR. The *Leishmania* sp. Name, GenBank accession number of first match by BLAST, species name, % identity, % coverage, and number of base pairs compared excluding the primer sequences, are included for each positive animal.

| **Animal number** | **ITS1 locus** | **kDNA minicircle** |
| --- | --- | --- |
| Dog 9020 | *L. infantum* (100%, 100%, MG969403.1, 184) | *L. infantum* (90%, 89%, KY699963.1, 59) |
| Dog 9021 | - | *L. infantum* (88%, 93%,KY699954.1, 63) |
| Dog 9026 | - | *L. infantum* (96%, 83%, AF190475.1, 54) |
| Dog 9029 | - | *L. infantum* (87%, 86%, KY699956.1, 65) |
| Dog 9035 | *L. infantum* (100%, 100%, MG969403.1, 184) | *L. infantum* (95%, 91%, KY699954.1, 64) |
| Dog 9036 | - | *L. infantum* (88%, 85%, KY699942.1, 67) |
| Dog 9040 | *L. infantum* (100%, 99%, MN648768.1, 186) | *L. infantum* (90%, 95%, MK770157.1, 64) |
| Dog 9044 | - | *L. infantum* (100%, 83%, KY699760.1, 51) |
| Dog 9046 | *L. infantum* (100%, 100%, MG969403.1, 184) |  |
| Dog 9047 | *L. infantum* (100%, 100%, MG969403.1, 184) | *L. infantum* (89%, 83%, KY699963.1, 65) |
| Dog 9050 | *L. infantum* (100%, 100%, MG969403.1, 184) |  |
| Dog 9067 | - | *L. infantum* (86%, 94%, MK770157.1, 67) |
| Dog 9074 | - | *L. infantum* (93%, 98%, MK770157.1, 63) |
| Dog 9078 | - | *L. infantum* (89%, 81%, EU370888.1, 66) |
| Dog 9079 | *L. infantum* (100%, 100%, MG969403.1, 184) | *L. infantum* (92%, 93%, KY699934.1, 65) |
| Cat 2 | - | *L. infantum* (94%, 92%, KY699971.1, 53) |
| Cat 14 | - | *L. infantum* (100%, 94%, KY699920.1, 57) |
| Cat 18 | - | *L. infantum* (100%, 100%, KY699971.1, 50) |
| Cat 25 | - | *L. infantum* (100%, 100%, KY699920.1, 50) |
| Cat 27 | - | *L. infantum* (100%, 100%,KY699971.1, 50) |
| Cat 36 | - | *L. infantum* (100%, 95%, KY699941.1, 53) |
| Cat 42 | - | *L. infantum* (100%, 100%, KY699920.1, 50) |
| Cat 46 | - | *L. infantum* (98%, 100%,KY699971.1, 54) |
| Cat 57 | - | *L. infantum* (98%, 96%, KY699956.1, 56) |
| Cat 61 | - | *L. infantum* (100%, 100%, KY699936.1,49) |
